# Description of a New Eyeless Cavefish Using Integrative Taxonomic Methods—*Sinocyclocheilus wanlanensis* (Cypriniformes, Cyprinidae), from Guizhou, China †

**DOI:** 10.3390/ani15152216

**Published:** 2025-07-28

**Authors:** Yewei Liu, Tingru Mao, Hiranya Sudasinghe, Rongjiao Chen, Jian Yang, Madhava Meegaskumbura

**Affiliations:** 1 Guangxi Key Laboratory for Forest Ecology and Conservation, College of Forestry, Guangxi University, Nanning 530004, China; liuyewei2017@outlook.com (Y.L.); cavefisher1996@gmail.com (T.M.); chenrongjiao2025@outlook.com (R.C.); 2 Division of Evolutionary Ecology, Institute of Ecology and Evolution, University of Bern, 3012 Bern, Switzerland; hsudasinghe@gmail.com; 3 Naturhistorisches Museum Bern, Bernastrasse15, 3005 Bern, Switzerland; 4 Key Laboratory of Environment Change and Resource Use, Beibu Gulf, Nanning Normal University, Nanning 530001, China; yj1981yj@163.com

**Keywords:** *Sinocyclocheilus*, new species, Beipanjiang River, *cytochrome b*, *NADH dehydrogenase subunit 4*, integrative taxonomy, cavefish, molecular systematics, mtDNA, China

## Abstract

China’s southwestern karst landscapes support remarkable cavefish diversity, especially within *Sinocyclocheilus*, the world’s largest cavefish genus. Using integrative taxonomic methods, we describe *Sinocyclocheilus wanlanensis* sp. nov., found in a subterranean river in Guizhou Province. This species lacks horn-like cranial structures; its eyes are either reduced to a dark spot or absent. It possesses a pronounced nuchal hump and a forward-protruding, duckbill-shaped head. Morphometric analysis of 28 individuals from six species shows clear separation from related taxa. Nano-CT imaging reveals distinct vertebral and cranial features. Phylogenetic analyses of mitochondrial *cytb* and *ND4* genes place *S. wanlanensis* within *S. angularis* group as sister to *S. bicornutus*, with p-distances of 1.7% (*cytb*) and 0.7% (*ND4*), consistent with sister-species patterns within the genus. *Sinocyclocheilus wanlanensis* is differentiated from *S. bicornutus* by its eyeless or degenerate-eye condition and lack of bifurcated horns. It differs from *S. zhenfengensis*, its morphologically closest species, in having degenerate or absent eyes, shorter maxillary barbels, and pelvic fins that reach the anus. The combination of morphological and molecular evidence supports its recognition as a distinct species. Accurate documentation of such endemic and narrowly distributed taxa is important for conservation and for understanding speciation in cave habitats.

## Simple Summary

The karst caves of southwest China are home to an extraordinary diversity of cavefish, especially those in *Sinocyclocheilus* group, the largest cavefish genus in the world. Using a combination of morphology and genetic analyses, we describe a new species, *Sinocyclocheilus wanlanensis*, found in an underground river in Guizhou Province. This fish is eyeless or degenerate-eyed, has no horn-like structures on its head (unlike some of its relatives), and features a large hump behind the head and a snout shaped like a duck’s bill. Measurement and comparison with similar species show that it is distinct morphologically. DNA analysis of two mitochondrial genes places it close to *S. bicornutus*, a related species; the genetic differences, while small, are consistent with what we observe between known sister species. *Sinocyclocheilus wanlanensis* is also distinct in appearance: it has degenerated eyes (dark spot) or no eyes (compared to the normal eyes of *S. bicornutus*); it also lacks the split horn found in *S. bicornutus*. It can be distinguished from the similar-looking *S. zhenfengensis* by its eyeless/degenerate-eye condition, shorter facial barbels, and longer pelvic fins. Identifying and describing new species is important for protecting cave life and understanding how species evolve in extreme environments.

## 1. Introduction

The extensive limestone landscapes of China, encompassing over 907,000 km^2^, stand among the largest karst distributions in the world [1,2]. The southwestern region, principally Yunnan, Guizhou, and Guangxi, spans more than 620,000 km^2^ of karst landforms, offering an ideal setting for cavefish evolution [3]. China has the most diverse array of cavefish species globally, with *Sinocyclocheilus*, the world’s most speciose cavefish genus, being the most prominent [4]. *Sinocyclocheilus* species exhibit diverse eye morphologies, categorized as normal, microphthalmic (micro-eyes), and anophthalmic (eyeless) [5]. They also possess cranial structures such as horns and nuchal humps to various degrees—this array of traits facilitates morphological analyses and species description. At present, genus *Sinocyclocheilus* contains 82 mostly stygomorphic species [6,7]. With the exception of *S. sanxiaensis* from the Three Gorges area, all species occur in the main karst zones of Guangxi, Guizhou, and Yunnan [2,8].

The history of *Sinocyclocheilus* studies dates back to 1904, when C. T. Regan first collected a specimen from Dianchi Lake in Yunnan. Initially identified as *Barbus grahami*, this specimen was later reclassified as *Sinocyclocheilus grahami* [9]. Further research in 1936 by Fang Bingwen led to the discovery of a new species in Fuxian Lake, Yunnan [10], which differed significantly from other cyprinid fishes. Consequently, Fang established this as the type for the genus, naming it *Sinocyclocheilus tingi* [11]. Early descriptions relied exclusively on morphological traits. Since the early 2000s, mitochondrial DNA has been integrated to delineate species (e.g., *cytochrome b*, *ND4*) [12]. More recently, genome-wide data have been applied to infer diversification patterns within the genus [13,14], though these data are yet to be used for species delimitation [15].

During our explorations, we collected specimens of a stygomorphic *Sinocyclocheilus* population in a southwestern karst area in Guizhou, whose eyes were absent or degenerated into dark spots. Here, we test whether this population represents a new species using an integrative approach involving morphological and molecular phylogenetic analyses.

Our analysis confirms that these specimens represent a previously undescribed lineage of *Sinocyclocheilus*, which we recognise as a new species. These results will contribute to our understanding of *Sinocyclocheilus* taxonomy, helping to clarify species diversity within this genus. Accurate species identification is crucial for conservation efforts and preserving China’s vulnerable karst ecosystems.

## 2. Materials and Methods

### 2.1. Specimen Sampling

**Figure 1:**
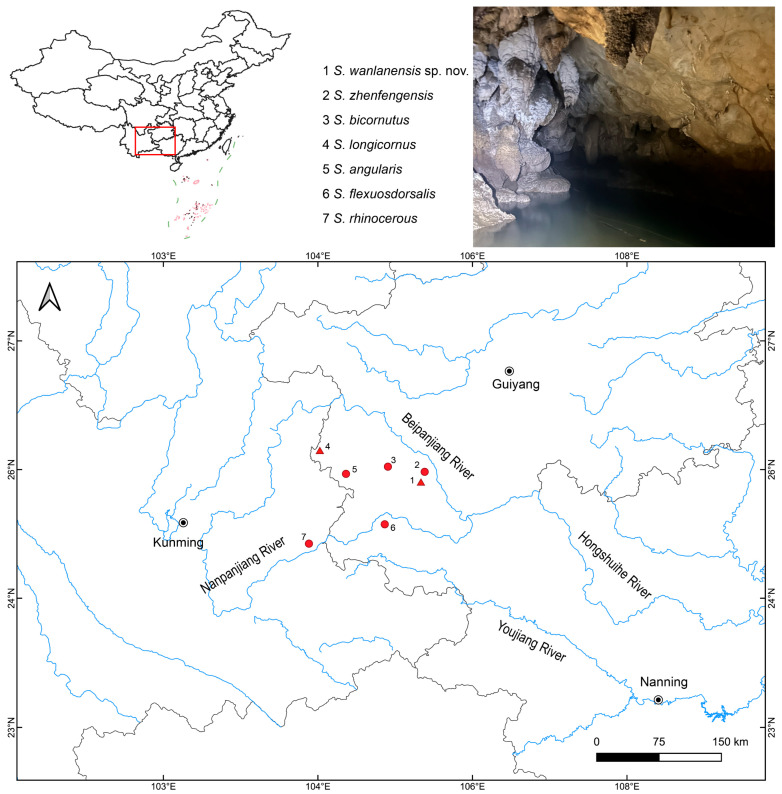
Sampling localities of *Sinocyclocheilus wanlanensis* and its relatives in this study. Triangles represent species that are eyeless, while circles represent species that have micro-eyes or normal eyes. Habitat photo of *S. wanlanensis* is in the inset. The red square indicates the region of *Sinocyclocheilus* distribution.

Between 2019 and 2024, we sampled *Sinocyclocheilus* species in Guizhou and Yunnan provinces (Figure 1). Depending on the cave type, we used different collection methods, including direct hand nets, trap nets, and, in a few instances, cave diving. The following species co-inhabited the Beipanjiang river system, or the distribution range is relatively close, where the new species was found: *S. longicornus* [16], *S. angularis* [17], *S. bicornutus* [18], *S. flexuosdorsalis* [19], *S. rhinocerous* [20], and *S. zhenfengensis* [21].

Due to the rarity and inaccessibility of deep cave habitats, only a limited number of specimens were collected for each species. In total, 28 *Sinocyclocheilus* specimens were obtained. These included three individuals representing an undescribed species from Wanlan Town, Zhenfeng County; *S. bicornutus* (*n* = 5) from Xiashan Town, Xingren City; *S. angularis* (*n* = 6) from Baotian Town, Panzhou City; *S. zhenfengensis* (*n* = 5) from Zhexiang Town, Zhenfeng County; *S. longicornus* (*n* = 6) from Hongguo Town, Panzhou City; *S. flexuosdorsalis* (*n* = 3) from Tianshengqiao Town, Longlin County; and *S. rhinocerous* (*n* = 1) from Wulong Village, Shizong County.

The live fish were first anesthetized with MS-222, and then the right pelvic fins of some fish were dissected and placed in 95% ethanol. These specimens were then placed in a 10% formaldehyde solution for fixation. Finally, formalin-fixed specimens were transferred to 75% ethanol for long-term preservation. All new specimens were deposited in Guangxi University (GXU), Nanning City, Guangxi Zhuang Autonomous Region; other comparative materials have been stored in Guizhou Normal University (GZNU), Yunyan District, Guiyang City, Guizhou Province, China.

### 2.2. Morphological Comparison

Morphometric data were collected from 28 preserved specimens of *Sinocyclocheilus*. We took each measurement 3 times with digital calipers, and then the average value was recorded to the nearest 0.1 mm, following published protocols [7,22,23]. Morphometric and meristic data were taken following the methods of published protocols [7,22] and provided in Table S1. The only specimen of *S. rhinocerous* collected by us was poorly preserved, and no morphometric or meristic data could be obtained. The lateral line scale count was taken from the upper margin of the operculum to the end of the caudal peduncle. Where lateral line scales were indistinct or absent, only the lateral line pores were counted. The last two branched rays of the dorsal and anal fins, when articulating on a single pterygiophore, were counted as one. All morphometric measurements were converted to standard length (SL) percentages, rounded to 0.1%, and subjected to a logarithmic transformation for morphometric analysis [24]. Because the new species lacks or has degenerate eyes, the four eye-related variables (snout length, eyeball diameter, eye diameter, and interorbital width) were excluded from the morphometric analysis. Despite the limited number of specimens, an exploratory principal component analysis (PCA) was conducted on the linear measurements to visualize general patterns of morphological variation among species and identify morphometric variables that differentiate species in multivariate space. The analysis was performed using the software PAST v.4.04 [25].

Nano-computed tomography (nano-CT) scanning and three-dimensional (3D) reconstruction of specimens representing the new species and its closely related species were performed using the Tomography and Digital Imaging system (GE phoenix v|tome|x m 300 & 180 CT) at the Key Laboratory of Vertebrate Origin and Human Evolution, Institute of Vertebrate Paleontology and Paleoanthropology (IVPP), Chinese Academy of Sciences. The entire CT scan of each specimen was conducted with an operating voltage of 80 kV and a current of 80 mA. Following a 360° rotational scan, the data were reconstructed into 1536 slices with 4096 × 4096 pixels and an image resolution of 12.5 µm. Virtual model reconstruction was performed using Volume Graphics Studio 3.4.0.

### 2.3. DNA Extraction, PCR and Sequencing

DNA was extracted from 95% ethanol-fixed fin tissue using the DNeasy Blood and Tissue Kit (Qiagen Inc., Valencia, CA, USA) following the manufacturer’s protocols. Fragments of the cytochrome b (*cytb*) and NADH dehydrogenase subunit 4 (*ND4*) genes were amplified by PCR (Polymerase Chain Reaction) using the primers DonThr R (5′-ACC TCC GAT CTT CGG ATT ACA AGA CCG-3′) and DonGlu F (5′-AAC CAC CGT TGT ATT CAA CTA CAA-3′) for *cytb* [26], ND4F (5′-AAC AAG ACC TCT GAT TTC GGC TCA-3′) and ND4R (5′-TAG CTT CCA CTT GGA TTT GCA CC-3′) for *ND4* [27].

Each PCR reaction, conducted in a 25 µL volume, consisted of 12.5 µL of the GoTaq^®^ Green Master Mix, 9.7 µL of nuclease-free water, 0.4 µL of each primer, and 2 µL of DNA extract. The PCR conditions for *cytb* followed an initial denaturation at 94 °C for 2 min, followed by 35 cycles of denaturation at 94 °C for 1 min, annealing at 48 °C for 1 min, extension at 72 °C for 1.5 min, and a final extension of 72 °C for 5 min; for *ND4*, an initial denaturation at 95 °C for 3 min, followed by 35 cycles of denaturation at 94 °C for 0.5 min, annealing at 51 °C for 0.5 min, extension at 72 °C for 1.5 min, and a final extension of 72 °C for 8 min [27,28]. PCR products were visualized by electrophoresis on a 1.0% agarose gel, then purified using a PCR purification kit (Qiagen), and sequenced in both directions with the corresponding primers by a commercial sequencing company. All newly generated sequences have been submitted to GenBank (Table S2).

### 2.4. Phylogenetic Analyses

We used a total of 246 mitochondrial gene sequences for molecular analyses (128 *cytb* sequences and 118 *ND4* sequences). The 240 *Sinocyclocheilus* sequences were downloaded from GenBank. We selected *Linichthys laticeps* and *Cyprinus carpio* as the outgroup (Table S2).

All sequences were adjusted manually and aligned with MAFFT [29] using ‘-auto’ strategy and normal alignment mode in PhyloSuite v.1.2.3 [30]. Alignment results were checked by eye and manually trimmed. The complete concatenated dataset included 128 samples of 65 *Sinocyclocheilus* species with a total alignment length of 2142 bp. Phylogenetic trees were constructed using maximum likelihood (ML) and Bayesian inference (BI) methods. The best-fitting nucleotide substitution model was estimated for each dataset according to the Bayesian Information Criterion with Partition Model using ModelFinder v.1.6.8 [31] as implemented by PhyloSuite v.1.2.3. The first, second, and third codon models of *cytb* and *ND4* genes were defined within Partition Model. The ML tree was conducted in IQ-TREE v.1.6.8 [32] as implemented by PhyloSuite v.1.2.3 with 5000 ultrafast bootstrap replicates and with the selected TPM2+I+G4, HKY+F+I+I+R2, and TIM2+F+I+G4 models for the first, second, and third codons of the dataset. Bayesian Inference was performed in MrBayes v.3.2.6 [33] under the selected K2P+I+G4, HKY+F+I+G4, and GTR+F+I+G4 models for three codons, using the MCMC method (24 chains simultaneously run for 1 × 10^7^ generations) to calculate posterior probability, with tree sampling frequency set to 1 per 1000 cycles and the initial 25% of the sampled data discarded as burn-in, resulting in a potential scale reduction factor of <0.01. Nodes in the trees were considered well supported when Bayesian posterior probabilities were ≥0.95 and the ML ultrafast bootstrap value was ≥95%. Uncorrected p-distances (1000 replicates) based on *cytb* and *ND4* genes were calculated using MEGA v.11.0 [34].

## 3. Results

### 3.1. Morphological Analyses

Table 1 summarizes the major diagnostic characters for *S. wanlanensis* and related species. Morphometric measurements of type specimens have been transferred to standard length (SL) percentage, as summarized in Table 2.

**Figure 2:**
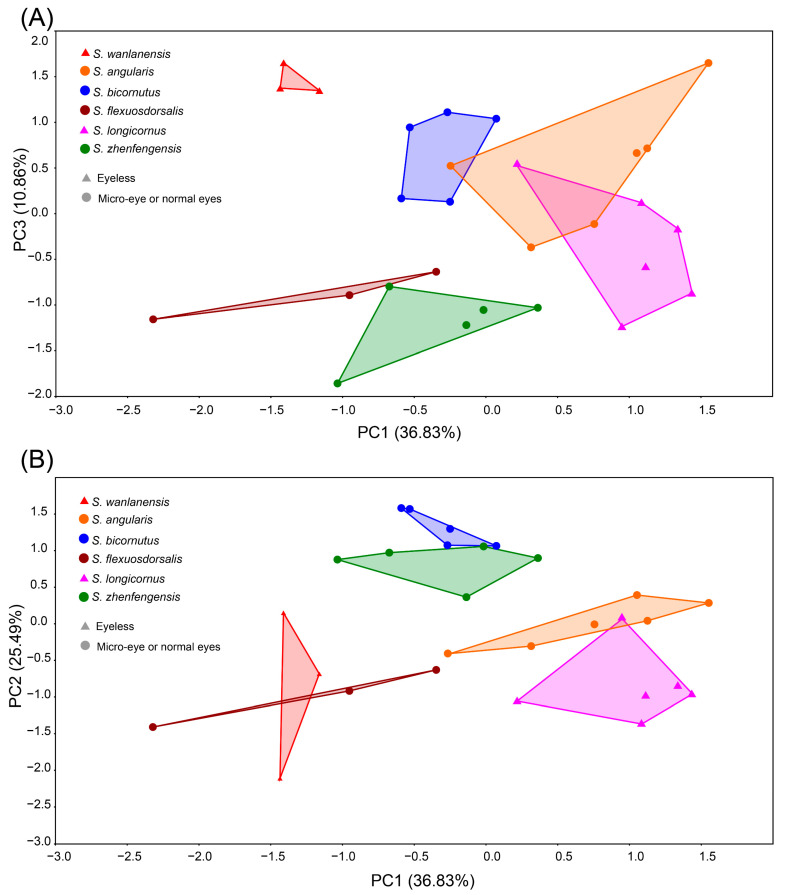
Results of principal component analysis (PCA) of overall body shape variation based on linear data along (**A**) PC1 vs. PC3 and (**B**) PC1 vs. PC2.

PCA of the dataset comprising 28 specimens from 6 species, based on 24 logarithmically transformed variables, revealed that the first 3 principal components accounted for 73.18% of the total variance. Specifically, PC1 explained 36.83%, PC2 25.49%, and PC3 10.86% of the variation (Figure 2). In the plot of PC1 vs. PC3, *S. wanlanensis* formed a distinct cluster along the PC3 axis, clearly separated from the other species included in the analysis (*S. angularis*, *S. bicornutus*, *S. flexuosdorsalis*, *S. longicornus*, and *S. zhenfengensis*) along the PC1 axis (Figure 2A). In contrast, the plot of PC1 vs. PC2 showed partial overlap between *S. flexuosdorsalis* and *S. wanlanensis*, although these two species clustered separately from the remaining four (*S. zhenfengensis*, *S. bicornutus*, *S. angularis*, and *S. longicornus*) (Figure 2B). Variables with high loadings on PC3 included lower jaw length, mouth width, dorsal fin base length, and pelvic fin length. Variables with high loadings on PC2 included pectoral fin base length, pelvic fin base length, mouth width, and the distance between the posterior nostrils. For PC1, maxillary barbel length and rictal barbel length contributed strongly.

**Figure 3:**
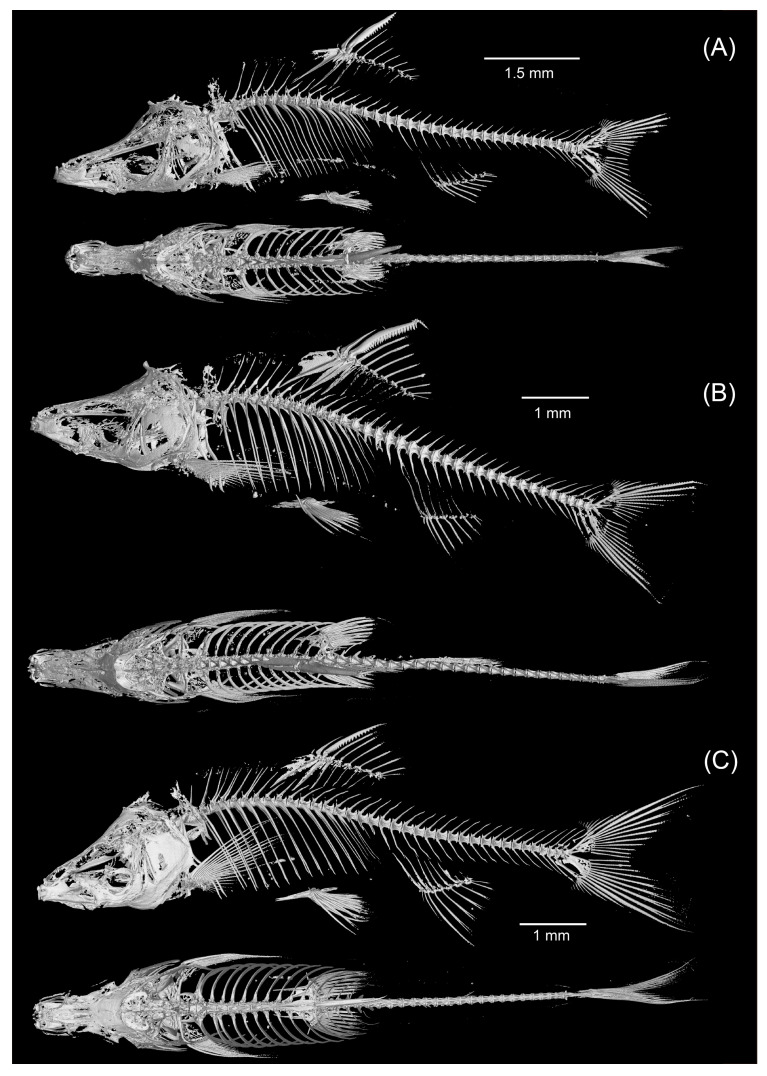
Micro-CT graph and reconstruction of (**A**) *S. wanlanensis*, (**B**) *S. bicornutus*, and (**C**) *S. zhenfengensis*.

CT imaging revealed that *S. wanlanensis*, *S. bicornutus*, and *S. zhenfengensis* exhibited a vertebral formula of 4 + 35 (Figure 3), indicating a shared pattern lacking diagnostic utility. The scans also showed that *S. wanlanensis* and *S. bicornutus* possessed prominent bony protrusions above the frontal bones. In contrast, such protrusions were not discernible in *S. zhenfengensis* (Figure 3C).

### 3.2. Phylogenetic Analyses and Genetic Divergence

**Figure 4:**
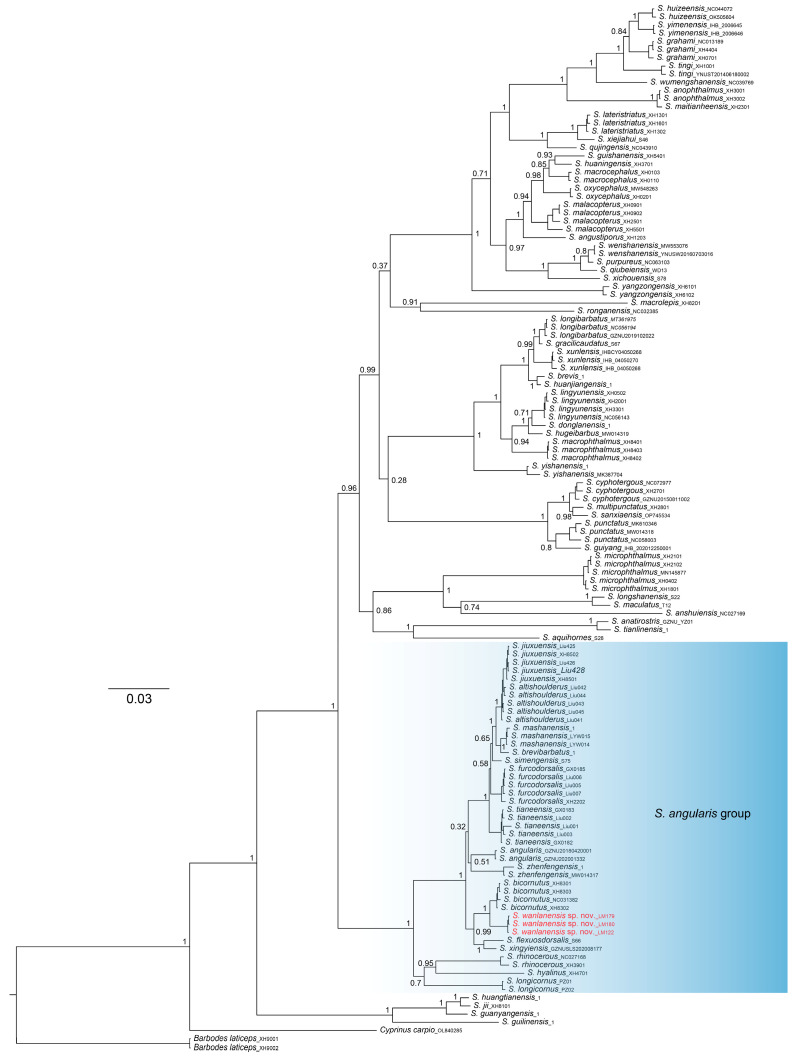
Molecular phylogenetic relationship of *Sinocyclocheilus*, based on Bayesian inference of *cytb* + *ND4* concatenated dataset. The numbers close to nodes represent Bayesian posterior probability. Red OTUs represent the lineage being described as a new species.

ML and BI phylogenies were constructed based on two concatenated mitochondrial gene sequences: *cytb* (1110 bp) and *ND4* (1032 bp). The resulting ML and BI trees exhibited similar overall topology, with only minor branching patterns and length differences (Figure 4 and Figure S1). Based on its phylogenetic position, the new species, *S. wanlanensis*, is placed within *S. angularis* group [11,15]. The monophyly of *Sinocyclocheilus* was strongly supported in both analyses. In both the ML and BI trees, *S. wanlanensis* formed a highly supported clade with its sister species *S. bicornutus* (ML bootstrap = 99; BI posterior probability = 1.00). Despite this concordance, some topological differences were evident between the two phylogenies. In the BI tree, *S. wanlanensis*, *S. bicornutus*, *S. xingyiensis* [35], and *S. flexuosdorsalis* formed a sister clade to a group comprising *S. jiuxuensis* [36], *S. altishoulderus* [37], *S. mashanensis* [38], *S. brevibarbatus* [39], *S. simengensis* [40], *S. furcodorsalis* [41], *S. tianeensis* [42], *S. angularis*, and *S. zhenfengensis*. In contrast, the ML tree placed *S. angularis* and *S. zhenfengensis* as sister to the clade comprising *S. jiuxuensis*, *S. altishoulderus*, *S. mashanensis*, *S. brevibarbatus*, *S. simengensis*, *S. furcodorsalis*, *S. tianeensis*, *S. wanlanensis*, *S. bicornutus*, *S. xingyiensis*, and *S. flexuosdorsalis*.

The uncorrected p-distances between *S. wanlanensis* and *S. bicornutus* (the genetically closest species) and *S. zhenfengensis* (the morphologically closest species) were 1.7% and 3.6%, respectively, based on *cytb* gene. For *ND4* gene, the corresponding p-distances were 0.7% and 2.4%, respectively (Tables S3 and S4).

### 3.3. Taxonomic Account

*Sinocyclocheilus wanlanensis* Liu, Mao & Yang, sp. nov.

**Figure 5:**
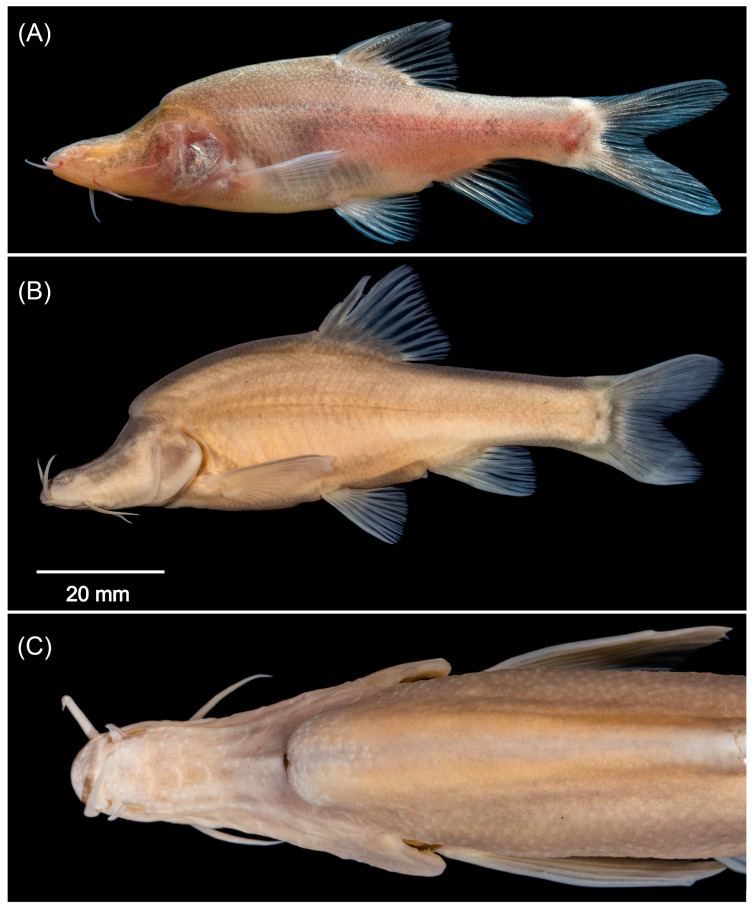
*Sinocyclocheilus wanlanensis* sp. nov., GXU2020000062, holotype, 86.74 mm SL. (**A**) Live specimen (not holotype); (**B**) lateral view of head in preserved specimen; (**C**) dorsal view of preserved specimen.

Figure 5, Table 1 and Table 2.

**Holotype**.

GXU2020000062, holotype, 86.7 mm SL; China: Guizhou: an underground river in a cave of Beipanjiang River of Pearl River Basin, Wanlan town, Zhenfeng county, 25.3617° N, 105.6131° E, altitude 905 m above sea level; by Yewei Liu, Chenghai Fu and Shipeng Zhou, on 14 November 2020.

**Paratypes**.

GXU2020000060 and GXU2020000061, 65.8–78.6 mm SL (two specimens), same data as the holotype.

**Diagnosis**.

*Sinocyclocheilus wanlanensis* is distinguished from all its congeners by the following combination of characters: absence of horn-like structure; eyes absent or degenerated into dark spots; a distinct nuchal hump; predorsal profile distinctly arched; tip of adpressed rostral barbel extending posteriorly not reaching vertical through anterior margin of sunken eye or dark spot; tip of pelvic-fin rays reaching anus when pelvic-fin rays extended backward; a distinct head shape protruding forward, resembling a duck’s beak; body scaleless; in life, body light golden brown; lateral line pores 41–45; gill rakers well developed, 9 on first gill arch.

**Species description**.

Meristics and proportional measurements are provided in Table 1 and Table 2. Body laterally compressed. Greatest body depth immediately anterior to origin of dorsal fin. Dorsal profile of head straight anteriorly; concave posteriorly. Predorsal profile of body convex with a distinct hump along back of head and then sloped toward dorsal-fin insertion. Postdorsal profile of body concave. Ventral profile of head straight. Ventral profile of body slightly convex between pectoral and pelvic-fin origins; straight between pelvic and anal-fin origins; slightly concave thereafter (Figure 5).

Head slightly compressed; laterally elongated; blunt in dorsal view. Mouth subterminal. Two pairs of barbels. Rostral barbel shorter than maxillary; extends slightly beyond origin of rostral barbel when adpressed but not reaching anterior margin of sunken eye or dark spot. Maxillary barbel when adpressed end before posterior edge of preoperculum. Eyes degenerate (dark spots) or absent.

Dorsal fin with 3 unbranched and 7 branched rays. Last unbranched ray stiff; posterior margin strongly serrated. Origin of dorsal fin slightly posterior to pelvic-fin origin; distal margin straight. Anal fin with 3 unbranched and 5 branched rays; distal margin straight. Origin of anal fin slightly closer to pelvic-fin origin than caudal-fin base. Pelvic fin with single unbranched and 7 branched rays. Origin of pelvic fin slightly closer to anal-fin origin than pectoral-fin origin, tip of pelvic-fin rays reaching anus when pelvic-fin rays extended backward. Pectoral fin with single unbranched and 14 (1), 15 (2) branched rays. Tip of pectoral fin when adpressed, reach beyond origin of dorsal fin vertically. Caudal fin with 8 + 8 branched rays, forked, lobes subequal, rounded distally.

Scales in body absent. Lateral-line pores 41, 43, 45.

**Colouration**.

In live specimens, head and body light golden brown. Barbels red. In preservative, head and body greyish white. Barbels white (Figure 5B,C).

**Distribution and habitat**.

Known only from a subterranean river within a cave in the town of Wanlan, Zhenfeng County, Guizhou Province, China. This underground river serves as an important source of drinking and irrigation water for local villagers. They have established large and small pumps to extract water from the cave. The water from this underground river eventually flows into the Beipanjiang River. *Sinycyclocheilus wanlanensis* occurs in sympatry with several other fish species: *Longanalus macrochirous*, *Pterocryptis anomala*, *Carassius auratus*, and *Opsariicthys bidens*.

**Etymology**.

The new species’ name, wanlanensis, derives from Wanlan Town, Zhenfeng County, where type specimens were collected.

## 4. Discussion

Morphological comparison and phylogenetic analysis support the distinct species status of *S. wanlanensis*. The species exhibits several key morphological traits that differentiate it from all other *Sinocyclocheilus* species: the eyes are absent or degenerated into dark spots; a pronounced nuchal hump; a distinctly arched predorsal profile; the tip of the adpressed rostral barbel extends posteriorly just beyond the vertical line through the anterior margin of the sunken eye or dark spot; and a distinct head shape that protrudes forward, resembling a duck’s beak.

**Figure 6:**
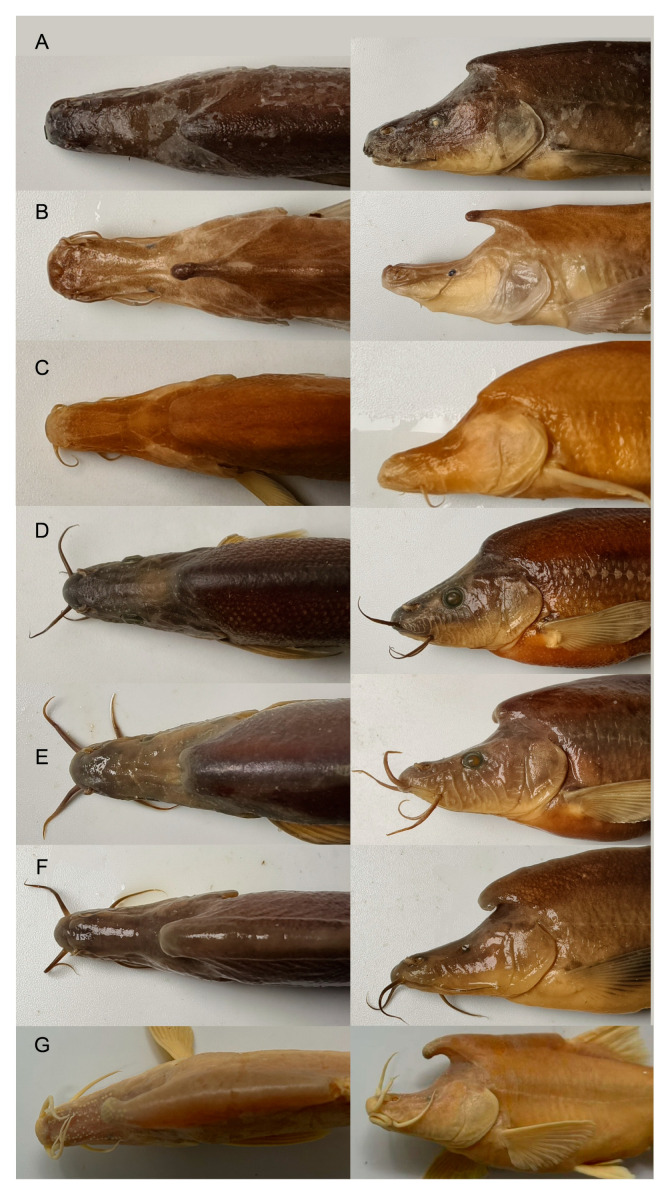
Lateral view and dorsal view of (**A**) *S. angularis*, GXU2020000063, 98.05 mm SL; China: Pearl River Basin; (**B**) *S. rhinocerous*, GXU2020000067, 110.1 mm SL; China: Pearl River Basin; (**C**) *S. wanlanensis*, GXU2020000060, 65.8 mm SL; China: Pearl River Basin; (**D**) *S. zhenfengensis*, GXU2020000024, 79.9 mm SL; China: Pearl River Basin; (**E**) *S. bicornutus*, GXU2020000007, 103.28 mm SL; China: Pearl River Basin; (**F**) *S. flexuosdorsalis*, GXU2020000066, 92.7 mm SL; China: Pearl River Basin; (**G**) *S. longicornus*, GZNU20210503005, 106.4 mm SL; China: Pearl River Basin.

Among the 16 species within *S. angularis* group, *S. wanlanensis* can be distinguished from *S. broadihornes* [43], *S. tileihornes* [44], and *S. rhinocerous* by the absence or degeneration of their eyes into dark spots (vs. presence of micro-eyes) and a forked horn-like structure being absent (vs. a single horn) (Figure 6). It can be distinguished from *S. angularis* by the absence or degeneration of their eyes into dark spots (vs. normal eyes) and the absence of a horn-like structure (vs. a single horn). It can be distinguished from *S. longicornus* by the absence of a horn-like structure (vs. a single and long horn). Furthermore, it differs from *S. xingyiensis* by the absence of eyes or degeneration into dark spots (vs. normal eyes) and from *S. hyalinus* [45] by possessing a horn-like structure that is absent (vs. a single horn). It can be distinguished from *S. flexuosdorsalis* by the absence of eyes or their degeneration into dark spots (vs. micro-eyes), the tip of the rostral barbel not reaching the sunken eye or dark spot (vs. reaching the anterior margin of the eyes), and the absence of the horn-like structure (vs. a single horn-like structure) (Figure 6). It differs from *S. brevibarbatus*, *S. jiuxuensis*, *S. altishoulderus*, *S. mashanensis*, and *S. simengensis* by the absence of eyes or their degeneration into dark spots (vs. normal eyes). *S. wanlanensis* can be distinguished from *S. furcodorsalis* and *S. tianeensis* by the absence of a horn-like structure (vs. a forked horn-like structure).

*Sinocyclocheilus wanlanensis* differs from morphologically similar *S. zhenfengensis* in having eyes that are absent or degenerated into dark spots (vs. normal eyes), the tip of the rostral barbel not reaching the degenerate eye (vs. reaching the anterior margin of the eyes), the tips of the pelvic-fin rays reaching the anus when the pelvic-fin rays are extended backwards (vs. not reaching the anus), and variation in gill raker counts (9 vs. 7–8). Additionally, it has a maxillary barbel length of 9.4–10.3% SL (vs. 12.4–14.5% SL), a longer head length of 31.4–33.8% SL (vs. 28.3–29.4% SL), a longer prepectoral length of 30.7–35.5% SL (vs. 26.4–28.7% SL), and a dorsal fin length of 20.3–24.4% SL (vs. 17.3–18.9% SL).

It is also distinguishable from *S. bicornutus* by having eyes that are absent or degenerated into dark spots (vs. normal eyes), absence of a horn-like structure (vs. a forked horn-like structure) (Figure 6), tip of rostral barbel not reaching sunken eye or dark spot (vs. reaching anterior margin of eyes), tips of pelvic-fin rays reaching anus when pelvic-fin rays are extended backward (vs. not reaching anus), variation in gill raker counts (9 vs. 7), body being scaleless (vs. complete body scales), shorter anal fin base length of 8.4–9.2% SL (vs. 10.2–11.5% SL), and a shorter pectoral fin base length of 3.5–4.1% SL (vs. 5.1–5.4% SL).

Our phylogenetic analyses support the distinctiveness of *S. wanlanensis* as a well-supported, independent evolutionary lineage (Figure 4). The genetic distances observed for the mitochondrial markers used (*cytb* and *ND4*) are comparable to those between sister taxa in several recently described species, including *S. xingyiensis* and *S. flexuosdorsalis*, as well as *S. xiejiahuai*, *S. lateristriatus*, and *S. panzhouensis* [35,46,47] (Table S3).

It is now established that mitochondrial DNA (mtDNA) is subject to selective sweeps, which can obscure phylogenetic signal [48]. As such, integrative approaches combining morphology and mtDNA data are more effective for species delimitation in *Sinocyclocheilus* [6,7,46]. *Sinocyclocheilus wanlanensis* conforms to this broader pattern.

To improve resolution, we are undertaking further analyses using genome-wide markers [15]. Restriction site-associated DNA sequencing (RAD-seq), in particular, has been shown to provide higher resolution for delineating independent lineages within *Sinocyclocheilus* [14,15,48]. Preliminary RAD-seq results support the distinctiveness of *S. wanlanensis* and also reveal additional cryptic diversity not evident in the mtDNA phylogenies [15]. This suggests that evolutionary constraints on mtDNA may limit its utility in resolving relationships among taxa inhabiting extreme cave environments.

## 5. Conclusions

The evidence from this integrative analysis, which combines detailed morphological, morphometric, and molecular data, supports the species status of *S. wanlanensis*. Its distinct morphology, characterized by absent or degenerated eyes that appear as dark spots and specific fin and body features, is complemented by its phylogenetic separation from closely related and morphologically similar species. The genetic divergence observed in *cytb* and *ND4* genes reinforces this taxonomic placement. Therefore, *S. wanlanensis* represents a well-defined species within *Sinocyclocheilus* genus, contributing to understanding cavefish diversity and evolutionary adaptations in this unique karstic landscape.

## 6. Comparative Material

*Sinocyclocheilus wanlanensis*, 3 specimens, GXU2020000060-62, 65.8–86.7 mm SL; China: Guizhou Province: Wanlan Town, Zhenfeng County, Qianxinan Buyei and Miao Autonomous Prefecture; by Yewei Liu, Chenghai Fu and Shipeng Zhou, on 14 November 2020. These specimens are stored at Guangxi University, East Daxue Road, Xixiangtang District, Nanning, Guangxi, China.

*Sinocyclocheilus bicornutus*, 5 specimens, GXU2020000005-09, 90.4–103.3 mm SL; China: Guizhou Province: Xiashan Town, Xingren City; by Yewei Liu, Chenghai Fu and Shipeng Zhou, on 20 May 2020. These specimens are stored at Guangxi University, East Daxue Road, Xixiangtang District, Nanning, Guangxi, China.

*Sinocyclocheilus angularis*, 6 specimens, GXU2020000063, GZNU20210505001, GZNU20210505003-04, GZNU20210505006-07, 74.2–98.1 mm SL; Panzhou City, Guizhou Province, China: Guizhou Province: Baotian Town, Panzhou City; by Tao Luo, Jiajun Zhou, and Xingliang Wang, on 5 May 2021. These specimens are stored at Guangxi University, East Daxue Road, Xixiangtang District, Nanning, Guangxi, China and Guizhou Normal University, Yunyan District, Guiyang City, Guizhou Province, China.

*Sinocyclocheilus zhenfengensis*, 5 specimens, GXU2020000023-27, 79.9–97.8 mm SL; China: Guizhou Province: Zhexiang Town, Zhenfeng County, Qianxinan Buyei and Miao Autonomous Prefecture; by Yewei Liu, Chenghai Fu and Shipeng Zhou, on 14 November 2020. These specimens are stored at Guangxi University, East Daxue Road, Xixiangtang District, Nanning, Guangxi, China.

*Sinocyclocheilus longicornus*, 6 specimens, GZNU20210503005, GZNU20210503009-11, GZNU20210503013, GZNU20210503015, 85.9–106.4 mm SL; China: Guizhou Province: Hongguo Town, Panzhou City; by Tao Luo, Jiajun Zhou, and Xingliang Wang, on 6 May 2021. These specimens are stored at the Guizhou Normal University, Yunyan District, Guiyang City, Guizhou Province, China.

*Sinocyclocheilus flexuosdorsalis*, 3 specimens, GXU2020000056-58, 85.6–92.7 mm SL; China: Guangxi Zhuang Autonomous Region: Tianshengqiao Town, Longlin County, Baise City; by Yewei Liu, Chenghai Fu and Shipeng Zhou, on 8 July 2024. These specimens are stored at Guangxi University, East Daxue Road, Xixiangtang District, Nanning, Guangxi, China.

*Sinocyclocheilus rhinocerous*, 1 specimen, GXU2020000067; China: Yunnan Province: Wulong Village, Shizong County; by Yewei Liu and Chenghai Fu, on 9 August 2019. This specimen is stored at Guangxi University, East Daxue Road, Xixiangtang District, Nanning, Guangxi, China.

## Figures and Tables

**Table 1:** Meristic comparison of morphological characteristics among *Sinocyclocheilus wanlanensis* and its related species.

	*S. wanlanensis*	*S. bicornutus*	*S. zhenfengensis*	*S. angularis*	*S. flexuosdorsalis*	*S. longicornus*
**Source**	This study	This study	This study	This study	This study	This study
**No. of specimens**	3	5	5	6	3	6
**Location**	Beipanjiang	Beipanjiang	Beipanjiang	Beipanjiang	Hongshuihe	Beipanjiang
**Dorsal fin rays**	iii, 7	iii, 7	iii, 6–7	iii, 7	iii, 7–8	iii, 7–8
**Anal fin rays**	iii, 5	iii, 5	iii, 5	iii, 5	iii, 5	iii, 5
**Pectoral fin rays**	i, 14–15	i, 14–15	i, 15	i, 14–17	i, 12–13	i, 14
**Pelvic fin rays**	i, 7	i, 6–7	i, 7	i, 7–8	i, 6–7	i, 7–8
**Lateral line scales/pores**	41–45	36–39	39–40	37–40	38–40	39–43
**Scale rows above lateral line**	/	7–11	8–9	/	11	/
**Scale rows below lateral line**	/	5–7	7–8	/	9–10	/
**Body scales**	Scaleless	Complete	Complete	Scaleless	Complete	Scaleless
**Eyes**	Blind(2) or Dark spots(1)	Normal eyes	Normal eyes	Normal eyes	Micro-eyes	Dark spots

**Table 2:** Morphological comparison of *S. wanlanensis*, *S. bicornutus*, *S. zhenfengensis*, *S. angularis*, *S. flexuosdorsalis,* and *S. longicornus*.

Measurements	*S. wanlanensis* (*n* = 3)	*S. bicornutus* (*n* = 5)		*S. zhenfengensis* (*n* = 5)		*S. angularis* (*n* = 6)		*S. flexuosdorsalis* (*n* = 3)	*S. longicornus* (*n* = 6)	
	Range	Range	Mean ± SD	Range	Mean ± SD	Range	Mean ± SD	Range	Range	Mean ± SD
Standard length (mm)	65.8–86.7	90.4–103.3	98.0 ± 6.2	79.7–97.8	87.5 ± 6.8	74.2–98.1	92.5 ± 9.3	85.6–92.7	85.9–106.4	94.5 ± 7.1
In % of standard length										
Body depth	25.6–29.0	28.8–32.6	30.2 ± 1.7	25.9–28.3	27.4 ± 1.1	22.5–30.9	28.9 ± 3.2	26.4–29.0	27.6–31.7	29.4 ± 1.7
Predorsal length	58.7–60.5	54.5–56.6	55.5 ± 0.7	57.3–58.2	57.7 ± 0.4	54.6–59.6	56.7 ± 2.0	54.6–57.2	56.8–57.8	57.1 ± 0.4
Dorsal fin base length	14.2–15.6	16.2–17.0	16.5 ± 0.4	12.7–15.3	14.0 ± 1.1	13.3–16.1	14.9 ± 1.0	17.2–18.9	13.6–16.6	14.9 ± 1.0
Dorsal fin length	20.3–24.4	18.8–21.1	19.9 ± 1.0	17.3–18.9	18.2 ± 0.6	19.0–23.4	20.5 ± 1.6	21.9–22.1	19.7–22.8	21.5 ± 1.1
Preanal length	69.5–74.6	69.1–70.8	70.1 ± 0.7	68.6–73.1	70.8 ± 1.8	72.0–79.3	75.3 ± 2.8	70.7–71.9	72.9–77.0	74.9 ± 1.5
Anal fin base length	8.4–9.2	10.2–11.5	10.8 ± 0.5	8.7–10.2	9.7 ± 0.6	8.5–11.3	10.0 ± 1.1	10.5–11.8	8.6–10.3	9.4 ± 0.6
Anal fin length	17.7–21.5	15.9–18.2	17.1 ± 0.9	14.6–16.8	15.9 ± 0.8	15.0–18.2	17.2 ± 1.2	18.1–21.3	17.1–21.1	19.6 ± 1.4
Prepectoral length	30.7–35.5	28.4–29.8	29.0 ± 0.5	26.4–28.7	27.9 ± 0.9	29.3–34.3	31.0 ± 1.8	28.4–32.4	30.3–32.8	31.3 ± 0.8
Pectoral fin base length	3.5–4.1	5.1–5.4	5.2 ± 0.1	4.3–5.0	4.6 ± 0.3	3.5–5.1	4.2 ± 0.6	4.3–5.1	3.5–5.0	4.2 ± 0.6
Pectoral fin length	21.9–24.6	19.9–25.7	22.7 ± 2.3	18.4–23.9	21.7 ± 2.3	20.8–25.6	23.5 ± 1.6	24.7–29.2	25.0–27.2	25.9 ± 0.8
Prepelvic length	50.2–57.1	48.5–50.3	49.6 ± 0.7	48.8–53.0	50.9 ± 1.8	51.3–55.2	53.3 ± 1.7	49.3–51.3	52.6–54.7	53.8 ± 0.9
Pelvic fin base length	4.0–5.9	5.5–6.3	5.8 ± 0.3	4.9–6.3	5.6 ± 0.5	4.2–5.5	4.7 ± 0.4	5.1–6.4	3.8–5.1	4.7 ± 0.5
Pelvic fin length	16.2–19.4	13.8–17.2	15.7 ± 1.4	12.0–15.3	13.9 ± 1.2	14.8–17.3	16.1 ± 1.0	17.9–20.3	15.9–18.5	17.2 ± 0.9
Caudal peduncle length	18.5–20.9	20.6–22.9	21.8 ± 0.8	19.5–23.1	21.1 ± 1.3	16.4–19.1	18.4 ± 1.0	19.7–20.4	18.3–21.3	20.0 ± 1.1
Caudal peduncle depth	10.3–12.4	11.9–13.3	12.5 ± 0.6	13.1–13.6	13.4 ± 0.2	9.7–12.5	11.6 ± 1.0	12.7–13.9	10.6–12.3	11.4 ± 0.7
Head length	31.4–33.8	28.6–30.2	29.3 ± 0.6	28.3–29.4	29.0 ± 0.5	29.1–31.1	29.9 ± 0.7	29.5–31.2	29.2–30.6	30.0 ± 0.5
Head depth	14.8–16.5	14.7–16.3	15.6 ± 0.7	16.6–18.3	17.6 ± 0.7	14.7–16.6	15.9 ± 0.7	15.4–16.4	16.0–18.8	18.1 ± 1.1
Head width	14.2–16.2	14.1–16.2	15.1 ± 0.9	14.7–16.5	15.5 ± 0.6	12.9–15.0	14.0 ± 0.8	13.5–13.8	12.9–15.3	13.7 ± 0.9
Snout length	/	9.6–10.8	10.3 ± 0.5	8.6–10.4	9.7 ± 0.7	8.1–10.1	9.3 ± 0.7	9.7–10.7	/	/
Eyeball diameter	/	2.9–4.7	3.9 ± 0.9	4.6–4.9	4.7 ± 0.1	3.7–4.5	4.1 ± 0.3	2.4–3.2	/	/
Eye diameter	/	5.7–8.3	7.1 ± 1.1	7.6–8.7	8.1 ± 0.4	6.2–7.6	6.8 ± 0.5	5.4–6.8	/	/
Interorbital width	/	8.9–9.4	9.2 ± 0,2	9.8–11.2	10.6 ± 0.5	8.0–9.3	8.7 ± 0.5	6.0–6.3	/	/
Prenostril length	5.1–5.6	4.1–5.2	4.8 ± 0.4	5.4–6.1	5.6 ± 0.3	4.8–5.7	5.3 ± 0.4	4.9–5.9	4.3–5.6	5.1 ± 0.6
Width between posterior nostrils	4.9–6.4	6.2–7.1	6.7 ± 0.4	5.4–7.2	6.5 ± 0.7	4.4–5.5	5.1 ± 0.6	4.9–5.4	4.8–6.0	5.3 ± 0.4
Upper jaw length	8.3–8.8	7.7–8.9	8.4 ± 0.5	7.2–8.6	8.0 ± 0.5	7.1–9.1	7.8 ± 0.7	7.5–8.8	6.3–8.2	7.4 ± 0.7
Lower jaw length	7.7–8.2	5.6–6.7	6.3 ± 0.4	5.6–6.9	6.5 ± 0.6	6.0–7.2	6.3 ± 0.4	6.7–8.0	4.86–7.52	6.2 ± 1.0
Mouth width	5.3–7.1	7.8–8.1	7.9 ± 0.1	5.7–6.6	6.0 ± 0.4	4.6–7.1	6.0 ± 1.0	5.8–6.8	5.2–7.0	6.3 ± 0.7
Maxilla barbel length	9.4–10.3	10.7–13.3	11.9 ± 1.2	12.4–14.5	13.6 ± 0.9	7.2–16.0	12.7 ± 3.3	11.2–16.7	13.6–21.1	17.7 ± 2.4
Rictal barbel length	8.3–10.6	9.2–13.5	11.0 ± 1.6	9.7–14.9	11.9 ± 2.2	9.1–15.3	12.7 ± 2.6	12.8–17.4	12.6–17.4	15.7 ± 1.7

## Data Availability

The original genetic data presented in the study are openly available in NCBI. All other data are available within the Supplementary Materials of the paper.

## Supplementary Materials

The following supporting information can be downloaded at: https://www.mdpi.com/article/10.3390/ani15152216/s1, Figure S1: Molecular phylogenetic relationships of *Sinocyclocheilus*, based on maximum likelihood inference of the concatenated *cytb* + *ND4* dataset. The numbers near the nodes represent the ML bootstrap values, respectively; Table S1: Measurements of the adult specimens of *Sinocyclocheilus wanlanensis* and its relatives. All units in mm. * For the holotype, ^#^ branched rays; Table S2: Voucher information, and GenBank numbers for all samples used. -, not available; Table S3: Uncorrected p-distance (%) between 22 individuals of the genus *Sinocyclocheilus* based on mitochondrial *cytb*; Table S4: Uncorrected p-distance (%) between 19 individuals of the genus *Sinocyclocheilus* based on mitochondrial *ND4*.
