# Supplementary material for: Description of a New Eyeless Cavefish Using Integrative Taxonomic Methods—Sinocyclocheilus wanlanensis (Cypriniformes, Cyprinidae), from Guizhou, China
Source: Animals (Basel). 2025 Jul 28;15(15):2216. doi: 10.3390/ani15152216 (PMC12345476; doi:10.3390/ani15152216)
Supplement: Supplementary file 1 [file animals-15-02216-s001.zip › animals-3742780.Supplementary file_Revision1.pdf]

Table S1. Measurements of the adult specimens of *Sinocyclocheilus wanlanensis* and its relatives. All units in mm. * For the holotype, # branched rays.

Species	Voucher	Dorsal fin	Pectoral fin	Tail fin#	Anal fin	Pelvic fin	SL	BD	PL	DFL	DBL	PAL	ABL	AFL	PPTL	PTBL	PTFL	PPVL	PVBL
S. wanlanensis sp. nov.	GXU2020000060	iii, 7	i, 15	16	iii, 7	i, 7	65.8	19.1	38.8	10.3	13.3	45.7	5.8	14.2	20.4	2.3	14.4	33.0	3.3
S. wanlanensis sp. nov.	GXU2020000061	iii, 7	i, 13	16	iii, 7	i, 7	78.6	20.1	47.6	11.2	19.2	58.7	6.6	16.4	27.9	2.9	19.1	44.9	3.1
S. wanlanensis sp. nov.	GXU2020000062*	iii, 7	i, 15	16	iii, 7	i, 7	86.7	24.6	50.9	13.2	18.2	61.7	8.0	15.3	26.6	3.5	21.4	44.2	5.1
S. bicornutus	GXU2020000005	iii, 7	i, 15	17	iii, 5	i, 6	101.3	31.7	56.4	17.2	21.1	70.0	11.5	18.4	29.1	5.2	24.4	50.6	5.6
S. bicornutus	GXU2020000006	iii, 7	i, 15	17	iii, 5	i, 7	102.8	29.6	56.1	16.6	20.1	72.8	10.7	17.7	30.1	5.5	23.2	51.7	6.5
S. bicornutus	GXU2020000007	iii, 7	i, 14	17	iii, 5	i, 7	103.3	30.6	57.3	16.8	20.0	72.9	11.0	17.0	30.0	5.6	21.7	50.0	6.0
S. bicornutus	GXU2020000008	iii, 7	i, 14	17	iii, 5	i, 6	92.2	30.1	52.1	15.1	17.4	64.3	9.4	14.7	26.2	4.7	18.4	45.5	5.1
S. bicornutus	GXU2020000009	iii, 8	i, 15	17	iii, 5	i, 7	90.4	26.1	50.1	15.2	19.1	63.6	10.4	16.0	26.9	4.7	23.3	45.2	5.2
S. angularis	GXU2020000063	iii, 7	i, 14	16	iii, 5	i, 7	98.1	22.1	54.8	15.3	18.7	70.6	9.3	17.8	30.9	3.6	22.7	50.3	4.5
S. angularis	GZNU20210505001	iii, 7	i, 17	16	iii, 5	i, 8	98.0	30.3	53.5	14.8	20.0	77.7	10.8	17.9	29.5	3.4	23.1	54.1	4.3
S. angularis	GZNU20210505003	iii, 7	i, 15	16	iii, 5	i, 8	95.4	28.6	52.1	14.3	19.8	71.6	9.9	16.5	28.5	3.9	24.4	49.5	4.7
S. angularis	GZNU20210505004	iii, 7	i, 15	16	iii, 5	i, 7	97.5	28.1	56.7	14.0	22.8	75.4	9.2	16.8	33.5	3.9	23.8	53.8	5.3
S. angularis	GZNU20210505006	iii, 7	i, 15	16	iii, 5	i, 7	92.2	28.2	55.0	12.3	18.7	70.1	7.8	13.9	27.0	4.7	19.2	49.4	4.4
S. angularis	GZNU20210505007	iii, 7	i, 14	17	iii, 5	i, 8	74.2	22.8	42.5	11.9	14.2	53.6	8.4	12.9	22.9	3.5	17.5	38.8	3.1
S. zhenfengensis	GXU2020000023	iii, 7	i, 15	14	iii-5	i, 7	83.6	23.6	47.9	12.3	15.8	61.1	8.5	12.2	23.6	4.2	17.2	44.3	4.6
S. zhenfengensis	GXU2020000024	iii, 7	i, 15	16	iii-5	i, 7	79.9	21.2	46.2	11.2	13.8	55.5	8.1	12.8	21.1	3.5	14.7	39.0	5.0
S. zhenfengensis	GXU2020000025	iii, 7	i, 15	17	iii-5	i, 7	90.1	25.5	51.6	13.8	16.3	64.5	9.0	14.6	25.5	4.0	20.2	45.6	5.4
S. zhenfengensis	GXU2020000026	iii, 7	i, 14	17	iii-5	i, 7	86.2	22.4	50.0	11.0	15.9	59.1	8.3	13.8	24.0	4.1	20.6	42.8	4.8
S. zhenfengensis	GXU2020000027	iii, 7	i, 15	16	iii-5	i, 7	97.8	27.5	57.0	12.7	17.8	69.6	8.5	16.5	28.1	4.3	22.9	51.3	4.8
S. longicornus	GZNU20210503005	ii, 7	i, 14	16	iii-5	i, 6	106.4	33.7	60.4	15.8	21.0	80.2	10.9	18.2	33.1	5.0	27.3	56.0	5.5
S. longicornus	GZNU20210505009	ii, 7	i, 14	16	iii-5	i, 6	95.4	29.5	54.2	15.8	20.7	71.5	9.3	19.6	28.9	4.3	25.2	51.8	4.5
S. longicornus	GZNU20210505010	ii, 7	i, 14	15	iii-5	i, 6	92.5	25.7	53.5	14.4	20.2	69.8	8.6	18.1	30.3	3.6	23.5	50.6	4.3

S. longicornus	GZNU20210505011	ii, 7	i, 14	16	iii-5	i, 6	89.6	24.8	50.9	13.0	20.4	65.8	8.7	18.9	27.9	4.5	24.3	47.3	4.5
S. longicornus	GZNU20210505013	ii, 7	i, 13	16	iii-5	i, 6	85.9	25.5	49.3	12.4	18.9	62.6	7.4	17.3	26.9	3.1	22.0	46.2	3.2
S. longicornus	GZNU20210505015	ii, 7	i, 14	16	iii-5	i, 6	97.0	27.7	55.3	13.2	20.1	74.7	8.7	18.4	30.2	3.4	24.3	53.0	4.6
S. flexuosdorsalis	GXU2020000064	iii, 8	i, 13	17	iii-5	i, 7	86.0	24.9	47.5	15.5	18.9	61.1	9.4	15.9	26.0	4.4	24.4	42.4	5.5
S. flexuosdorsalis	GXU2020000065	iii, 7	i, 12	17	iii-5	i, 6	85.6	22.6	46.7	14.7	18.9	60.5	9.0	15.5	24.3	3.9	21.2	42.5	4.4
S. flexuosdorsalis	GXU2020000066	iii, 8	i, 13	17	iii-5	i, 7	92.7	26.7	53.0	17.6	20.3	66.6	10.9	19.8	30.1	4.0	27.1	47.5	5.4

Table S2. Voucher information, and GenBank numbers for all samples used. -, not available.

Taxon	*Cytb* voucher number	*ND4* voucher number	Accession No.		Source
			Cytb	*ND4*	
S. altishoulderus_Liu041	Liu041	Liu041	PQ505198	PQ505222	NCBI
S. altishoulderus_Liu042	Liu042	Liu042	PQ505199	PQ505223	NCBI
S. altishoulderus_Liu043	Liu043	Liu043	PQ505200	PQ505224	NCBI
S. altishoulderus_Liu044	Liu044	-	PQ505201	-	NCBI
S. altishoulderus_Liu045	Liu045	Liu045	PQ505202	PQ505225	NCBI
S. anatirostris_GZNU_YZ01	GZNU_YZ01	GZNU_YZ01	NC069226	NC069226	NCBI
S. angularis_GZNU20180420001	GZNU20180420001	GZNU20180420001	MZ636514	MZ636514	NCBI
S. angularis_GZNU202001332	GZNU202001332	GZNU202001332	MW362289	MW362289	NCBI
S. angustiporus_XH1203	XH1203	XH1203	AY854702	AY854759	NCBI
S. anophthalmus_XH3001	XH3001	XH3001	AY854715	AY854772	NCBI
S. anophthalmus_XH3002	XH3002	XH3002	AY854716	AY854773	NCBI
S. anshuiensis_NC027169	-	-	NC027169	NC027169	NCBI
S. aquihornes_S28	S28	S28	PQ155086	PQ155094	NCBI
S. bicornutus_NC031382	-	-	NC031382	NC031382	NCBI
S. bicornutus_XH8301	XH8301	XH8301	AY854730	AY854787	NCBI
S. bicornutus_XH8302	XH8302	XH8302	AY854731	AY854788	NCBI
S. bicornutus_XH8303	XH8303	XH8303	AY854732	AY854789	NCBI
S. brevibarbatus_1	GX0064-L20-13	GX0066	MT373106	MW548423	NCBI
S. brevis_1	GX0155	LIU108	MT373105	MW548424	NCBI
S. wanlanensis_LM179	LM179	LM179	PV817858	PV817861	This study
S. wanlanensis_LM180	LM180	LM180	PV817859	PV817862	This study
S. wanlanensis_LM122	LM122	LM122	PV817857	PV817860	This study
S. cyphotergous_GZNU20150811002	GZNU20150811002	GZNU20150811002	MW024370	MW024370	NCBI
S. cyphotergous_NC072977	-	-	NC072977	NC072977	NCBI
S. cyphotergous_XH2701	XH2701	XH2701	AY854711	AY854768	NCBI
S. donglanensis_1	CA141	LIU050	AB196441	MW548425	NCBI
S. flexuosdorsalis_S66	S66	-	OQ718397	-	NCBI
S. furcodorsalis_GX0185	GX0185	GX0185	PQ505208	PQ505231	NCBI
S. furcodorsalis_LIU005	LIU005	LIU005	PQ505209	PQ505232	NCBI
S. furcodorsalis_LIU006	LIU006	LIU006	PQ505210	PQ505233	NCBI
S. furcodorsalis_LIU007	LIU007	LIU007	PQ505211	PQ505234	NCBI
S. furcodorsalis_XH2202	XH2202	XH2202	AY854709	AY854766	NCBI
S. gracilicaudatus_S67	S67	-	OQ718398	-	NCBI
S. grahami_NC013189	-	-	NC013189	NC013189	NCBI
S. grahami_XH0701	XH0701	XH0701	AY854694	AY854751	NCBI
S. grahami_XH4404	XH4404	XH4404	AY854696	AY854753	NCBI
S. guanyangensis_1	GX0173	LIU016	MT373108	MW548426	NCBI
S. guilinensis_1	GX0073	LIU057	MT373104	MW548427	NCBI
S. guishanensis_XH5401	XH5401	XH5401	AY854722	AY854779	NCBI

S. guiyang_IHB_202012250001	IHB_202012250001	IHB_202012250001	OR141734	-	NCBI
S. huangtianensis_1	GX0175	LIU129	MT373109		NCBI
S. huaningensis_XH3701	XH3701	XH3701	AY854718	AY854775	NCBI
S. huanjiangensis_1	GX0124	LIU087	MT373103	MW548429	NCBI
S. hugeibarbus_MW014319	-	-	MW014319	MW014319	NCBI
S. huizeensis_NC044072	-	-	NC044072	NC044072	NCBI
S. huizeensis_OK505604	-	-	OK505604	OK505604	NCBI
S. hyalinus_XH4701	XH4701	XH4701	AY854721	AY854778	NCBI
S. jii_XH8101	XH8101	XH8101	AY854727	AY854784	NCBI
S. jiuxuensis_Liu425	Liu425	Liu425	PQ505212	PQ505235	NCBI
S. jiuxuensis_Liu426	Liu426	Liu426	PQ505213	PQ505236	NCBI
S. jiuxuensis_Liu428	Liu428	Liu428	PQ505214	PQ505237	NCBI
S. jiuxuensis_XH8501	XH8501	XH8501	AY854736	AY854793	NCBI
S. jiuxuensis_XH8502	XH8502	XH8502	AY854737	AY854794	NCBI
S. lateristriatus_XH1301	XH1301	XH1301	AY854704	AY854761	NCBI
S. lateristriatus_XH1302	XH1302	XH1302	AY854705	AY854762	NCBI
S. lateristriatus_XH1601	XH1601	XH1601	AY854707	AY854764	NCBI
S. lingyunensis_NC056143	-	-	NC056143	NC056143	NCBI
S. lingyunensis_XH0502	XH0502	XH0502	AY854691	AY854748	NCBI
S. lingyunensis_XH2001	XH2001	XH2001	AY854692	AY854749	NCBI
S. lingyunensis_XH3301	XH3301	XH3301	AY854693	AY854750	NCBI
S. longibarbatus_GZNU2019102022	GZNU2019102022	GZNU2019102022	MW024371	MW024371	NCBI
S. longibarbatus_MT361975	-	-	MT361975	MT361975	NCBI
S. longibarbatus_NC056194	-	-	NC056194	NC056194	NCBI
S. longicornus_PZ01	PZ01	-	MZ634123	MZ634125	NCBI
S. longicornus_PZ02	PZ02	-	MZ634124	MZ634126	NCBI
S. longshanensis_S22	S22	S22	PQ155085	PQ155093	NCBI
S. macrocephalus_XH0103	XH0103	XH0103	AY854683	AY854740	NCBI
S. macrocephalus_XH0110	XH0110	XH0110	AY854684	AY854741	NCBI
S. macrolepis_XH8201	XH8201	XH8201	AY854729	AY854786	NCBI
S. macrophthalmus_XH8401	XH8401	XH8401	AY854733	AY854790	NCBI
S. macrophthalmus_XH8402	XH8402	XH8402	AY854734	AY854791	NCBI
S. macrophthalmus_XH8403	XH8403	XH8403	AY854735	AY854792	NCBI
S. maculatus_T12	T12	-	MF325008	-	NCBI
S. maitianheensis_XH2301	XH2301	XH2301	AY854710	AY854767	NCBI
S. malacopterus_XH0901	XH0901	XH0901	AY854697	AY854754	NCBI
S. malacopterus_XH0902	XH0902	XH0902	AY854698	AY854755	NCBI
S. malacopterus_XH2501	XH2501	XH2501	AY854699	AY854756	NCBI
S. malacopterus_XH5501	XH5501	XH5501	AY854700	AY854757	NCBI
S. mashanensis_1	GX0026	LIU066	MT373107	MW548430	NCBI
S. mashanensis_LYW014	LYW014	LYW014	PQ505215	PQ505238	NCBI
S. mashanensis_LYW015	LYW015	LYW015	PQ505216	PQ505239	NCBI
S. microphthalmus_MN145877	-	-	MN145877	MN145877	NCBI

S. microphthalmus_XH0402	XH0402	XH0402	AY854687	AY854744	NCBI
S. microphthalmus_XH1801	XH1801	XH1801	AY854688	AY854745	NCBI
S. microphthalmus_XH2101	XH2101	XH2101	AY854689	AY854746	NCBI
S. microphthalmus_XH2102	XH2102	XH2102	AY854690	AY854747	NCBI
S. multipunctatus_XH2801	XH2801	XH2801	AY854712	AY854769	NCBI
S. oxycephalus_MW548263	-	-	MW548263	MW548263	NCBI
S. oxycephalus_XH0201	XH0201	XH0201	AY854685	AY854742	NCBI
S. punctatus_MK610346	-	-	MK610346	MK610346	NCBI
S. punctatus_MW014318	-	-	MW014318	MW014318	NCBI
S. punctatus_NC058003	-	-	NC058003	NC058003	NCBI
S. purpureus_NC063103	-	-	NC063103	NC063103	NCBI
S. qiubeiensis_WD13	WD13	-	MF324996	-	NCBI
S. qujingensis_NC043910	-	-	NC043910	NC043910	NCBI
S. rhinocerous_NC027168	-	-	NC027168	NC027168	NCBI
S. rhinocerous_XH3901	XH3901	XH3901	AY854720	AY854777	NCBI
S. ronganensis_NC032385	-	-	NC032385	NC032385	NCBI
S. sanxiaensis_OP745534	-	-	OP745534	OP745534	NCBI
S. simengensis_S75	S75	-	OQ718406	-	NCBI
S. tianeensis_GX0182	GX0182	GX0182	PQ505217	PQ505240	NCBI
S. tianeensis_GX0183	GX0183	GX0183	PQ505218	PQ505241	NCBI
S. tianeensis_LIU001	LIU001	LIU001	PQ505219	PQ505242	NCBI
S. tianeensis_LIU002	LIU002	LIU002	PQ505220	PQ505243	NCBI
S. tianeensis_LIU003	LIU003	LIU003	PQ505221	PQ505244	NCBI
S. tianlinensis_1	GX0087	GX0121	MT373102	MW548431	NCBI
S. tingi_XH1001	XH1001	XH1001	AY854701	AY854758	NCBI
S. tingi_YNUST201406180002	YNUST201406180002	YNUST201406180002	NC039594	NC039594	NCBI
S. wenshanensis_MW553076	-	-	MW553076	MW553076	NCBI
S. wenshanensis_YNUSW20160703016	YNUSW20160703016	YNUSW20160703016	NC060737	NC060737	NCBI
S. wumengshanensis_NC039769	-	-	NC039769	NC039769	NCBI
S. xichouensis_S78	S78	-	OQ718409	-	NCBI
S. xiejiahuai_S46	S46	S46	PQ165088	PQ165088	NCBI
S. xingyiensis_GZNUSLS202008177	GZNUSLS202008177	-	ON573218	-	NCBI
S. xunlensis_IHBCY04050268	IHBCY04050268	IHBCY04050268	HM536791	HM536710	NCBI
S. xunlensis_IHB_04050268	IHB_04050268	IHB_04050268	EU366187	EU366184	NCBI
S. xunlensis_IHB_04050270	IHB_04050270	IHB_04050270	EU366190	EU366185	NCBI
S. yangzongensis_XH6101	XH6101	XH6101	AY854725	AY854782	NCBI
S. yangzongensis_XH6102	XH6102	XH6102	AY854726	AY854783	NCBI
S. yimenensis_IHB_2006645	IHB_2006645	IHB_2006645	EU366192	EU366179	NCBI
S. yimenensis_IHB_2006646	IHB_2006646	IHB_2006646	EU366191	EU366180	NCBI
S. yishanensis_1	GX0070	LIU095	MT373101	MW548432	NCBI

S. yishanensis_MK387704	-	-	MK387704	MK387704	NCBI
S. zhenfengensis_1	-	-	MK610342	MK610347	NCBI
S. zhenfengensis_MW014317	-	-	MW014317	MW014317	NCBI
Barbodes laticeps_XH9001	XH9001	XH9001	AY854738	AY854795	NCBI
Barbodes laticeps_XH9002	XH9002	XH9002	AY854739	AY854796	NCBI
Cyprinus carpio_OL840285	qingtian1	qingtian1	OL840285	OL840285	NCBI

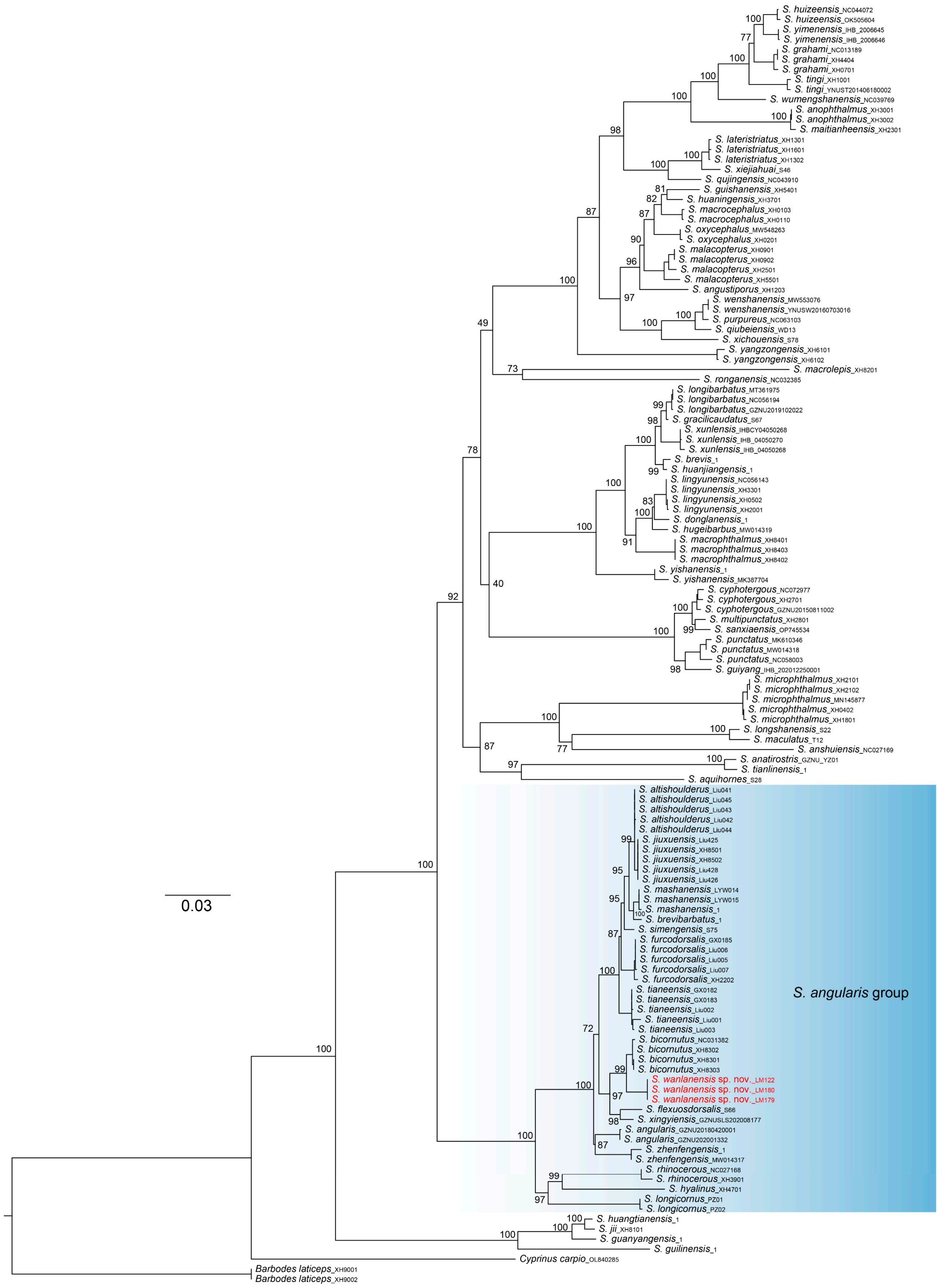

Figure S1. Molecular phylogenetic relationship of *Sinocyclocheilus*, based on maximum likelihood of the *cytb* + *ND4* concatenated data set. The numbers close to nodes represent the ML bootstrap values, respectively.
